# Clinical decision-making in remote rheumatology consultations: a service evaluation of new patient and inflammatory rheumatic disease follow-up appointments

**DOI:** 10.1093/rap/rkab036

**Published:** 2021-06-04

**Authors:** Ahmed B Tarar, Jake Weddell, Fay Manning, Shouma Dutta, Zoe Paskins, Ian C Scott

**Affiliations:** 1 Haywood Academic Rheumatology Centre, Haywood Hospital, Midlands Partnership NHS Foundation Trust, Burslem; 2 Primary Care Centre Versus Arthritis, School of Medicine, Keele University, Keele, UK

Key message

- Many remote rheumatology consultations lead to in-person reviews, particularly for newly referred patients.

Dear Editor, Owing to coronavirus disease 2019 (COVID-19), remote appointments have largely replaced face-to-face consultations [1]. Although important to reducing viral spread, they have been implemented rapidly, despite clinicians/patients having limited remote consultation experience [2]. Consequently, clinicians may feel reluctant to make diagnoses/escalate immunosuppression without seeing patients in person, leading to many remote consultations converting to face-to-face reviews. This is particularly true for newly referred patients, with many rheumatology clinicians avoiding the use of remote appointments for new patients [3].

To date, research on the effectiveness of remote rheumatology consultations is limited mainly to established conditions, with trained presenters examining patients [4]. Only one UK-based study has described unselected remote consultation outcomes in the COVID-19 pandemic [5]; it did not report new and follow-up consultations separately.

Understanding in whom remote consultations are most effective is an issue of crucial importance; if many newly referred patients require face-to-face reviews, an argument could be made for offering them these initially. To address this knowledge gap, we conducted an evaluation of our remote service at the Haywood Hospital (Stoke-on-Trent) to understand how often/why new-patient and follow-up patient consultations converted to face-to-face reviews. We also evaluated how often newly referred patients received a diagnosis/treatment change/were discharged and how often patients with established inflammatory arthritis/vasculitis/CTD diagnoses received alterations in immunosuppression.

We evaluated all newly referred patients and follow-up patients with diagnoses of inflammatory arthritis/CTD/vasculitis, aged ≥18 years and receiving remote consultations during April–June 2020 (new referrals: all weeks; follow-up patients: second weeks). Data were extracted retrospectively from clinic letters. For all consultations, we recorded whether they converted to a face-to-face consultation for their next review and the reason/time frame for this. For new referrals, we recorded the indication, diagnosis made, whether treatment was initiated and whether patients were discharged. For follow-up consultations, we recorded the diagnosis, whether their disease was considered active (based on the clinician’s opinion in the letter); if active, whether they were offered an increase in DMARD/CS treatment and whether they accepted this; and if inactive, whether they were offered a decrease in DMARD treatment and whether they accepted this. Data were analysed descriptively using R (v.4.0.3) and summarized as means/proportions. The evaluation was registered with our trust’s service evaluation department. Ethical approval was not required.

Three hundred and thirteen new-patient consultations occurred (303 telephone; 10 telephone and video). The mean patient age was 53.7 (95% CI: 51.7, 55.6) years; 207 (66.1%) were female, and 274 (87.5%) of White British ethnicity. Commonest referral reasons comprised arthralgia [93 (29.7%) patients], suspected synovitis [90 (28.8%) patients] and suspected CTD [52 (16.6%) patients]. Definite and probable/possible diagnoses were made in 38 (12.1%) and 227 (72.5%) patients, respectively. Of these, the commonest diagnoses were OA, RA, FM, CTD and peripheral SpA [41 (15.5%), 36 (13.6%), 32 (12.1%), 30 (11.3%) and 26 (9.8%) patients, respectively]. In 48 (15.3%) patients, no diagnosis was made. Fifty-five (17.6%) patients were discharged, in whom the commonest diagnoses comprised OA, FM and a positive ANA without associated features of a rheumatological condition [14 (25.5%), 10 (18.2%) and 9 (16.4%) patients, respectively]. Ninety-six (30.7%) patients received drug treatment (19 a DMARD; 25 a CS; 51 an analgesic).

**Fig. 1:**
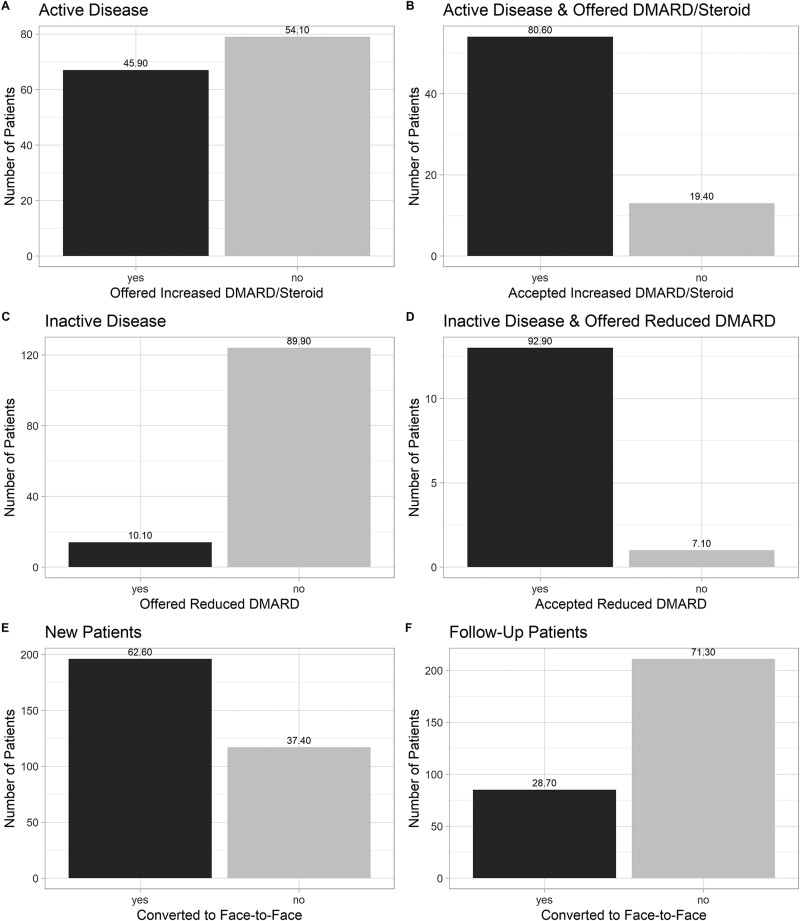
Bar charts of treatment decisions and face-to-face consultation conversions in patients receiving remote consultations For all bar charts, the percentage of patients is provided at the top of the bar. (**A**) People with inflammatory arthritis/CTD/vasculitis felt to have active disease and offered an increase in immunosuppression. (**B**) People with inflammatory arthritis/CTD/vasculitis felt to have active disease and offered an increase in immunosuppression who accepted this increase. (**C**) People with inflammatory arthritis/CTD/vasculitis felt to have inactive disease and offered a decrease in immunosuppression. (**D**) People with inflammatory arthritis/CTD/vasculitis felt to have inactive disease and offered a decrease in immunosuppression who accepted this decrease. (**E**) People receiving a new-patient remote consultation converting to a face-to-face review. (**F**) People receiving a follow-up remote consultation converting to a face-to-face review.

Two hundred and ninety-six follow-up appointments occurred (293 telephone; 3 telephone and video), with two patients seen twice over the time period. The mean patient age was 63.2 (95% CI: 61.5, 64.9) years; 188 (63.9%) were female, and 284 (96.6%) of White British ethnicity. The commonest condition was RA [175 (59.5%) patients], followed by undifferentiated inflammatory arthritis [41 (13.9%) patients], CTD [31 (10.5%) patients], vasculitis [24 (8.2%) patients], peripheral SpA [15 (5.1%) patients] and axial spondyloarthritis [8 (2.7%) patients]. In 146 (49.3%) and 138 (46.6%) consultations, patients were felt to have active and inactive disease, respectively (disease activity unclear in 12). In those with active disease, 67 (45.9%) were offered an escalation in DMARDs/CSs, 54 of whom accepted this (Fig. 1). In those with inactive disease, 14 (10.1%) were offered a reduction in DMARDs, 13 of whom accepted this.

Nearly two-thirds [196 (62.6%)] of new-patient appointments converted to face-to-face consultations for their next review (Fig. 1); the reason in 192 of these was to perform an examination. Most [113 (57.7%)] were scheduled to occur as soon as possible/within 1 month. Nearly one-quarter [85 (28.7%)] of follow-up appointments converted to a face-to-face consultation, of which 69 were to perform an examination to establish disease activity. Twenty-eight (32.9%) were scheduled to occur as soon as possible/within 1 month.

Our evaluation, the chief limitation of which is that it evaluated data from a single centre, limiting generalizability, has three findings. First, given that nearly two-thirds of new-patient consultations converted to face-to-face reviews to conduct examinations, remote approaches seem better placed to deliver care to people with established diagnoses. Second, although clinicians feel able to assess disease activity remotely, they appear reluctant to alter immunosuppression in the pandemic setting, despite many patients being agreeable to alterations in immunosuppression. Third, a substantial proportion (28.3%) of patients with inflammatory arthritis/vasculitis/CTD continue to require face-to-face examinations to confirm disease activity. Many patient-reported outcome measures have been validated to assess disease activity in people with inflammatory arthritis [6, 7]. We consider that the feasibility and merits of using these to inform decision-making and deliver treat-to-target strategies [8] remotely requires urgent evaluation.
